# Effectiveness of Virtual Reality Training in Teaching Personal Protective Equipment Skills: A Randomized Clinical Trial

**DOI:** 10.1001/jamanetworkopen.2023.55358

**Published:** 2024-02-14

**Authors:** Keisuke Tsukada, Youichi Yasui, Satoshi Miyata, Junko Fuyumuro, Tomomi Kikuchi, Takuhiro Mizuno, Satoshi Nakayama, Hirotaka Kawano, Wataru Miyamoto

**Affiliations:** 1 Department of Orthopaedic Surgery, Teikyo University School of Medicine, Kaga Itabashi-ku, Tokyo, Japan; 2 Department of Infection Control, Teikyo University Hospital, Kaga Itabashi-ku, Tokyo, Japan; 3 Teikyo University Graduate School of Public Health, Kaga Itabashi-ku, Tokyo, Japan; 4 Alpha Code Inc, Toranomon Minato-ku, Tokyo, Japan

## Abstract

**Importance:**

Training on the proper use of personal protective equipment (PPE) is critical for infection prevention among health care workers. Traditional methods, such as face-to-face and video-based training, can strain resources and present challenges.

**Objective:**

To determine the effectiveness of 360° virtual reality (VR) training for PPE donning and doffing compared with face-to-face and video training in enhancing the PPE use skills of prospective health care practitioners.

**Design, Setting, and Participants:**

A blinded, prospective, and randomized noninferiority clinical trial was conducted from August to December 2021 at Teikyo University School of Medicine in Tokyo, Japan, with a mixed population of medical students. Participants were second- to fourth-year medicine, medical technology, or pharmacy students aged 20 years or older with no prior PPE training. Participants were randomized into 1 of 3 training groups (VR, face-to-face, or video) based on their enrollment order. An intention-to-treat analysis was conducted.

**Intervention:**

A 30-minute lecture on PPE procedures was delivered to all participants before the training. After the lecture, the VR group trained with an immersive 360° VR tool, the face-to-face group trained with actual PPE, and the video group trained by watching video footage on a computer and a projector. After 3 days, a standardized practical skills test was administered.

**Main Outcomes and Measures:**

The primary outcome was the mean score on a 20-point practical skills test, and the secondary outcome was the percentage of correct execution.

**Results:**

A total of 91 participants were recruited and randomized into 3 groups: VR (n = 30), face-to-face (n = 30), and video (n = 31) training. After excluding 1 participant due to illness, 90 participants (mean [SD] age, 24.2 [3.15] years; 54 males [60.0%]) completed the assessment. The mean (SD) scores were 17.70 (2.10) points for the VR group, 17.57 (2.45) points for the face-to-face group, and 15.87 (2.90) points for the video group. The VR group demonstrated no significant difference in performance from the face-to-face group. However, the VR group had significantly higher effectiveness than the video group (17.70 vs 15.87 points; *P* = .02).

**Conclusions and Relevance:**

Results of this trial indicate that VR training was as effective as face-to-face training in enhancing PPE donning and doffing skills and was superior to video training. The findings suggest that VR training is a viable resource-conserving training option.

**Trial Registration:**

Japan Registry of Clinical Trials Identifier: jRCT103021029

## Key Points

**Question:**

Is virtual reality (VR) training effective in teaching donning and doffing of personal protective equipment (PPE) to prospective health care practitioners?

**Findings:**

In this randomized noninferiority clinical trial of 91 students, VR and face-to-face training significantly outperformed and were superior to video training. Participants in the video group demonstrated significantly less accuracy in glove removal, hand hygiene, gown removal, and gown roll up.

**Meaning:**

Findings of this study suggest that VR is a viable resource-conserving alternative approach to conventional training for PPE donning and doffing techniques.

## Teaser

This randomized clinical trial examines 3 methods for teaching personal protective equipment donning and doffing techniques, including virtual reality, face-to-face, and video training, to medical, medical technology, and pharmacy students.

## Introduction

The COVID-19 pandemic substantially affected global health.^1^ As of November 2023, more than 676 million cases of COVID-19 have been documented worldwide, with approximately 6.88 million deaths.^2^ The transmission modes of COVID-19 include direct contact with respiratory droplets or aerosols from an infected person and indirect contact with surfaces or objects contaminated by the virus.^3^ Because of these transmission routes, the use of personal protective equipment (PPE) was an early priority during the pandemic, as the risk of infection was 12 times higher among health care workers compared with the general population.^4,5,6^ Accordingly, the correct donning and doffing of PPE was crucial to reducing infections and nosocomial transmission.^3,4,5,6,7,8^ Training for PPE donning and doffing traditionally involved face-to-face sessions using actual equipment. However, the pandemic caused a shortage of medical supplies, and physical distancing made face-to-face training difficult to implement.^9,10,11,12,13,14,15,16^

In recent years, advances in science and technology have revealed the usefulness of web- and video-based distance education as an alternative to traditional face-to-face instruction.^17,18^ This shift toward digital platforms has been particularly important in the field of medical education, wherein innovative methods are constantly explored to enhance learning and skill acquisition.^17,18^ Among these innovations, virtual reality (VR) training has emerged as an alternative to conventional face-to-face training in medical education. Virtual reality training is beneficial because it wastes no time and is not bound by time and space, and several studies have reported its effectiveness in knowledge and skill retention.^19,20,21,22,23^ Among the various types of VR, highly immersive forms constructed with computer graphics and 360° videos are commonly used. Several studies have shown the effectiveness of simulation-based training.^19,20,21,22,23,24^ Therefore, using VR to teach PPE donning and doffing techniques can be as effective as other forms of medical training.^25^ However, limited reports exist on VR training for PPE donning and doffing,^25^ and there is an even greater gap in comparative studies of VR training vs conventional face-to-face and video instruction in terms of efficacy and technique establishment. This scarcity of comparative data underscores the importance of the present research, which aimed to fill these gaps by evaluating and comparing the effectiveness of different training modalities. Consequently, we sought to provide a more comprehensive understanding of the most effective methods for teaching critical PPE donning and doffing skills, a crucial aspect of safety in medical practice. Thus, we aimed to determine the efficacy of 360° VR training, including an immersive 360° VR tool based on 360° imagery, for PPE donning and doffing compared with face-to-face and video training in enhancing the PPE use skills of prospective health care practitioners.

## Methods

### Study Design

A randomized noninferiority clinical trial with a prospective randomized open-blinded end point design was conducted at Teikyo University School of Medicine in Tokyo, Japan, between August and December 2021. The Teikyo University Medical Research Ethics Committee approved the trial. The trial protocol is provided in Supplement 1. Participants provided written informed consent. We followed the Consolidated Standards of Reporting Trials (CONSORT) reporting guideline.^26^

Eligible participants were second- to fourth-year medical, medical technology, and pharmacy students at Teikyo University School of Medicine. Recruitment was conducted through campus posters and announcements during lectures and clerkships. Students aged 20 years or older who volunteered to participate and had no prior PPE training were included in the study. Students who had experienced discomfort or adverse effects from VR or had difficulties with PPE donning and doffing were excluded. Participants were provided various options to choose their preferred date for the study. Both training workshops and practical examinations were held once a week. Written informed consent was obtained on a workshop day.

### Randomization and Blinding

**Figure 1.**
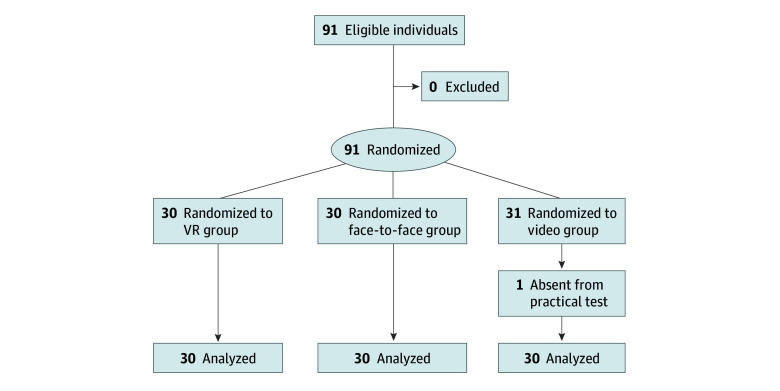
CONSORT Flow Diagram CONSORT indicates consolidated standards of reporting trials; VR, virtual reality.

Participants were randomized to 1 of 3 training groups: VR, face-to-face, or video (Figure 1). Randomization was based on the participants’ enrollment order using a sex-based randomization form. Stratified randomization minimized potential sex bias as it used an internet-based application to generate the allocation list.^27^ Prior to implementation, it was anticipated that the target sample population would have a greater number of males from the target population; therefore, stratified randomization based on sex was used to eliminate any bias between groups. Stratification by age and faculty affiliation was not performed during randomization.

To prevent biased evaluations, we concealed the group allocation of participants from the evaluators of the practical examinations (Y.Y., W.M.). Furthermore, we concealed the group identities from the statistical analyst (S.M.). Participants were informed of the randomization results on a workshop day and were grouped accordingly for practical training. Randomization was conducted the day before the workshop.

### Intervention

The study protocol is depicted in the eFigure in Supplement 2. Experienced instructors in the Department of Infection Control at Teikyo University Hospital delivered to all participants a standard 30-minute lecture using a common procedure manual on the proper technique for donning and doffing PPE. The lectures were conducted in groups of 5 to 15 participants to decrease transmission risk during the COVID-19 pandemic. After the lecture, the participants were divided into 3 groups: a VR group, which trained with an immersive 360° VR tool; a conventional face-to-face group, which trained with actual PPE; and a video group, which trained by watching video footage on a computer and a projector. The hands-on training lasted 30 minutes, and a single instructor supervised each group. A standardized protocol for PPE donning and doffing, based on guidelines from the Centers for Disease Control and Prevention, was established and followed. The VR group underwent 2 repetitions of the PPE donning and doffing procedures. Similarly, the face-to-face group performed 2 iterations of the procedures using actual PPE, whereas the video group viewed the training videos 3 times. The practical training was conducted in separate rooms to eliminate intergroup influence.

Three days after training, the participants’ practical PPE donning and doffing skills were evaluated through a standardized examination administered by the Department of Infection Control. The test was identical to the test taught during training and was completed individually, with each participant’s performance recorded on video from multiple perspectives. An expert evaluator analyzed the recordings to ensure the accuracy of the assessment. The evaluators were 2 experienced infection control specialists who have been conducting PPE donning and doffing training at the university for over 10 years. In terms of evaluation methods, we prevalidated the checklist and criteria for pass or fail scoring. After the practical test, the supervisor provided direct instruction to each participant on any inaccuracies in the donning and doffing procedures to ensure a correct understanding of the technique.

The immersive 360° VR tool used in this trial was developed in collaboration with Alpha Code Inc and was based on high-quality 8000 resolution 360° videos and recordings made with high-fidelity microphones. The tool is available for free to a broad audience in Japan and worldwide on a VR content distribution platform (Blinky; Alpha Code Inc).^28^ Users can choose from 3 perspectives (instructor, forward, or backward) and learn the correct and incorrect methods of donning and doffing PPE through a virtual instructor-led session.

The training materials for the video group were produced by the Department of Infection Control and have been used for many years in staff training at Teikyo University Hospital. The materials feature infection control experts performing the correct methods of PPE donning and doffing and explaining the key points of each procedure. The content of these training videos was identical to that used in the VR and face-to-face groups, ensuring consistency across all training modalities.

### Outcome Measures

The primary outcomes were the mean scores obtained from the PPE donning and doffing proficiency examination by the 3 groups. The secondary outcomes were the percentages of correct execution at each control step of the donning and doffing protocol among the 3 groups.

A total of 20 clinically essential skills were identified, of which 8 were for donning and 12 were for doffing (Table 1). One point was awarded for each skill performed correctly, with a maximum score of 20 points. The scoring criteria and a checklist were established by the Department of Infection Control. After the data collection, 2 evaluators (Y.Y., W.M.) who were blinded to the group allocation assessed a participant’s skills while viewing a video recording of the practical skill test. In the case of disagreements between the evaluators, an additional research member (S.N.), also blinded to the study group, was consulted to resolve the issue.

**Table 1.** Checklist for the Practical Skill Test

Checkpoint	Description
Donning PPE	
1	Perform hand hygiene
2	Sufficient hand hygiene was done
3	Put on a gown
4	The gown was put on correctly
5	Put on a surgical mask
6	The surgical mask was put on correctly
7	Put on gloves
8	The gloves were put on correctly
Doffing PPE	
1	Remove gloves
2	The gloves were removed correctly
3	Perform hand hygiene
4	Sufficient hand hygiene was done
5	Remove a gown
6	The gown sleeves were removed correctly
7	The gown roll up was correct
8	Remove a surgical mask
9	The surgical mask was removed correctly
10	Perform hand hygiene
11	Sufficient hand hygiene was done
12	The PPE disposal was accurate

Abbreviation: PPE, personal protective equipment.

### Sample Size Calculation

A biostatistician (S.M.) based the sample size on a pilot study conducted from January to February 2021 at Teikyo University School of Medicine. A standard 30-minute lecture on PPE donning and doffing and conventional face-to-face training using actual PPE were provided to 10 medical technology students. The pilot study students were first-time PPE donners and doffers. We and experienced educators in the Department of Infection Control conducted the lecture and face-to-face training. A practical examination was conducted 3 days after the workshop to assess the level of retention of the PPE donning and doffing technical procedure.

The preliminary study results indicated that the mean (SD) score of the practical examination for conventional face-to-face training was 17.70 (3.01) points. A clinically acceptable threshold of 16 points or higher was established, and the noninferiority margin was set at −1.70 points based on the difference between the pilot study result of 17.70 points and the acceptable standard of 16.00 points. The sample size for each group was calculated to be 16.30 for the primary outcome, with a 1-tailed significance level of *P* = .03, a power of 0.80, and an effect size of −1.70. This calculation was based on a 2-sample *t* test design for the primary end point as required for the noninferiority test. A technical skill sufficient for clinical practice was established as a mean score of 16 points and an effect size of −1.70 based on the preliminary study results. Additionally, 28.90 was the number of people calculated for each group for the secondary end points based on the estimated mean score (17.70 points for face-to-face, 16.00 points for VR, and 14.00 points for video groups), with a significance level of *P* = .01 and a power of 0.80 for each group. Hence, the number of samples required for the analysis of variance (ANOVA) was determined. Accordingly, the sample size for each group was set at 30 to account for potential cases deemed ineligible after enrollment.

### Statistical Analysis

An intention-to-treat analysis was conducted. Statistical analysis was performed by an experienced, nonaligned, blinded biostatistician (S.M.). Continuous data were quantified through the mean (SD) or median (IQR) scores, and categorical data were conveyed as frequencies (percentages). Comparisons between the 3 groups were performed with ANOVA, followed by Tukey honestly significant difference multiple comparisons. For individual evaluations between 2 groups, continuous data were subjected to an unpaired, 2-tailed *t* test for unequal variance and a Wilcoxon rank sum test. Categorical data underwent Fisher exact test, which was subsequently adjusted for multiplicity using the Holm method. Statistical analysis was conducted in January 2022 using R, version 4.2.2 (R Project for Statistical Computing).

## Results

A total of 91 participants were recruited and randomized into 3 groups: VR (n = 30), face-to-face (n = 30), and video (n = 31) training. After exclusion of 1 participant from the video group due to illness, 90 participants (mean [SD] age, 24.2 [3.15] years; 54 males [60.0%], 36 females [40.0%]) completed the assessment (Figure 1; Table 2). Most participants (75 [83.3%]) were affiliated with the Department of Medicine, while 3 (3.3%) were affiliated with the Department of Medical Technology and 12 (13.3%) were affiliated with the Department of Pharmacy. There were no significant differences in sex, age, or department affiliation among the 3 groups.

**Table 2.** Baseline Characteristics of Participants

Characteristic	Participants, No. (%)				*P* value
	All	VR group	Face-to-face group	Video group	
Total No. of participants	90	30	30	30	>.99
Sex					
Male	54 (60.0)	18 (60.0)	18 (60.0)	18 (60.0)	>.99
Female	36 (40.0)	12 (40.0)	12 (40.0)	12 (40.0)	>.99
Age, mean (SD), y	24.2 (3.15)	23.9 (2.33)	24.7 (3.79)	24.1 (4.13)	.64
Affiliated university department					
Medicine	75 (83.3)	23 (76.7)	26 (86.7)	26 (86.7)	.24
Medical technology	3 (3.3)	0	1 (3.3)	2 (6.7)	
Pharmacy	12 (13.3)	7 (23.3)	3 (10.0)	2 (6.7)	

Abbreviation: VR, virtual reality.

### Primary Outcome

**Figure 2.**
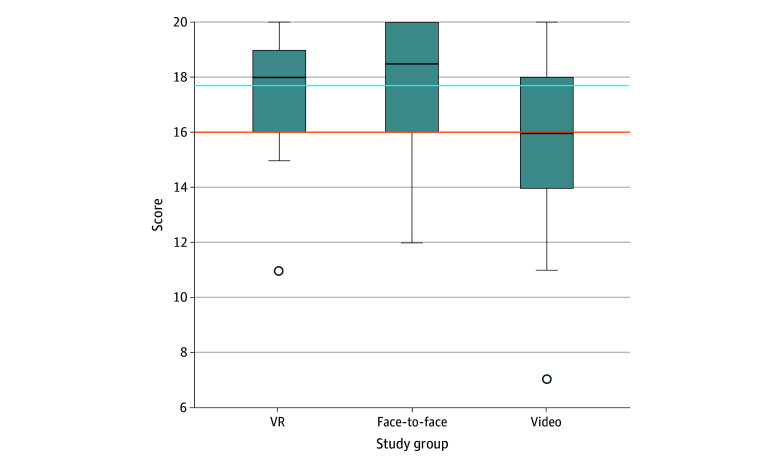
Practical Skill Test Results Upper and lower ends of the boxes represent third and first quartile, respectively; horizontal line inside boxes represents median; whiskers represent maximum and minimum scores; and circles below boxes represent minimum score of outliers. The orange line indicates clinically acceptable score, and the blue line indicates pilot study score. VR indicates virtual reality.

The overall mean (SD) score of the practical skills test was 17.04 (2.61) points. Each group had the following mean (SD) score: 17.70 (2.10) points for the VR group, 17.57 (2.45) points for the face-to-face group, and 15.87 (2.90) points for the video group (Figure 2). No adverse events were reported during the workshops or practical tests.

The mean difference between the VR and face-to-face groups was −0.133 (95% CI, −1.41 to –1.67; *P* = .98) after adjustment for multiplicity. This difference, along with the predefined noninferiority margin of −1.70, demonstrated that the VR group was not inferior to the face-to-face group in the practical skills test score (Figure 2).

The ANOVA showed a significant difference in learning effectiveness among the VR, face-to-face, and video training methods (17.70 vs 17.57 vs 15.87 points; *P* = .009). Further analysis revealed no significant difference between the VR and face-to-face groups. However, a significant decreasing pattern was observed between the video and VR groups (15.87 vs 17.70 points; *P* = .02) and between the video and face-to-face groups (15.87 vs 17.57 points; *P* = .03).

### Secondary Outcome

The correct execution of the donning steps was similar across all 3 groups (Table 3). Conversely, the doffing step results differed across the groups. The VR and face-to-face groups had similar good performances. In contrast, the video group had a significantly inferior accuracy rate in glove removal (22 [73.3%] vs 29 [96.7%] and 27 [90.0%]; *P* = .04), hand hygiene (19 [63.3%] vs 28 [93.3%] and 23 [76.7%]; *P* = .02), gown removal (9 [30.0%] vs 19 [63.3%] and 20 [66.7%]; *P* = .009), and gown roll up (6 [20.0%] vs 18 [60.0%] and 19 [63.3%]; *P* = .001) compared with the VR and face-to-face groups.

**Table 3.** Correct Execution of Each Checkpoint

Checkpoint	Participants, No. (%)				*P* value
	All (n = 90)	VR group (n = 30)	Face-to-face group (n = 30)	Video group (n = 30)	
Donning PPE					
Perform hand hygiene	90 (100)	30 (100)	30 (100)	30 (100)	>.99
Sufficient hand hygiene was done	76 (84.4)	27 (90.0)	25 (83.3)	24 (80.0)	.67
Put on a gown	89 (98.9)	30 (100)	30 (100)	29 (96.7)	>.99
The gown was put on correctly	88 (97.8)	30 (100)	30 (100)	28 (93.3)	.33
Put on a surgical mask	81 (90.0)	27 (90.0)	27 (90.0)	27 (90.0)	>.99
The surgical mask was put on correctly	86 (95.6)	29 (96.7)	30 (100)	27 (90.0)	.32
Put on gloves	81 (90.0)	27 (90.0)	27 (90.0)	27 (90.0)	>.99
The gloves were put on correctly	71 (78.9)	24 (80.0)	26 (86.7)	21 (70.0)	.32
Doffing PPE					
Remove gloves	89 (98.9)	30 (100)	30 (100)	29 (96.7)	>.99
The gloves were removed correctly	78 (86.7)	29 (96.7)	27 (90.0)	22 (73.3)	.04
Perform hand hygiene	83 (92.2)	29 (96.7)	28 (93.3)	26 (86.7)	.49
Sufficient hand hygiene was done	70 (77.8)	28 (93.3)	23 (76.7)	19 (63.3)	.02
Remove a gown	83 (92.2)	27 (90.0)	27 (90.0)	29 (96.7)	.69
The gown sleeves were removed correctly	48 (53.3)	19 (63.3)	20 (66.7)	9 (30.0)	.009
The gown roll up was correct	43 (47.8)	18 (60.0)	19 (63.3)	6 (20.0)	.001
Remove a surgical mask	81 (90.0)	26 (86.7)	27 (90.0)	28 (93.3)	.91
The surgical mask was removed correctly	74 (82.2)	25 (83.3)	26 (86.7)	23 (76.7)	.70
Perform hand hygiene	77 (85.6)	25 (83.3)	26 (86.7)	26 (86.7)	>.99
Sufficient hand hygiene was done	58 (64.4)	21 (70.0)	20 (66.7)	17 (56.7)	.62
The PPE disposal was accurate	88 (97.8)	30 (100)	29 (96.7)	29 (96.7)	>.99

Abbreviations: PPE, personal protective equipment; VR, virtual reality.

For a sensitivity analysis, we conducted multiple imputation for missing participants in the video group and obtained the results from the imputed data, which were similar to the original results. The results of ANOVA and multiple comparison were similar to the original results from the imputed data (VR and face-to-face groups: 17.70 and 17.57 [*P* = .98]; video and VR groups: 15.87 and 17.70 [*P* = .02]; and video and face-to-face groups: 15.87 vs 17.57 [*P* = .03]). The imputation method was predictive mean matching with 20 multiple imputations and 50 iterations. Hence, no bias was introduced to the results from the absence of participants.

Subgroup analysis based on faculty affiliation revealed no significant differences in performance between the VR and face-to-face groups within Teikyo University School of Medicine. Despite the lack of significant differences between the VR and video groups or between the face-to-face and video groups, likely due to sample size limitations, the *P* values were small, suggesting a pattern consistent with the overall findings. In the faculties of the Department of Pharmacy and Department of Medical Technology, sample sizes were insufficient to detect definitive patterns.

## Discussion

This trial evaluated the effectiveness of immersive 360° VR training for PPE donning and doffing skills and compared this approach with traditional face-to-face and video training methods. The findings indicated no significant difference in practical skills test scores between the VR and face-to-face groups; however, both groups significantly outperformed the video group. During the practical training, the video group had a significantly inferior accuracy rate in glove removal, hand hygiene, gown removal, and gown roll up. We anticipated that this trial would ascertain the effectiveness of VR training in PPE donning and doffing procedures. We believe these findings could be instrumental to training health care workers, particularly in circumstances wherein face-to-face training is not feasible, such as in a pandemic.

To our knowledge, only 1 study has investigated the application of VR to PPE.^25^ A comparative study by Kravitz et al^25^ found no significant difference in the effectiveness of PPE donning and doffing between game-style VR training and e-learning. Similar to the findings of Kravitz et al,^25^ results of the present study support the efficacy of an immersive 360° VR tool in PPE donning and doffing procedures. Within the medical field, there are 2 main types of VR: immersive 360° VR and game-style VR using computer graphics.^19,20,21,22,23,24^ Immersive 360° VR may offer equivalent or superior learning effectiveness compared with game-style VR for 2 principal reasons. First, a VR system using 360° videos can be developed at a reduced cost compared with game-style VR, a factor that underscores the benefit of broader generalization favoring immersive 360° VR. Previous literature has reported that the development of immersive 360° VR at a cost of US $25 000, while the cost of developing a game-style VR exceeded $100 000.^19,29^ Second, immersive 360° VR might be more conducive to learning detailed motions, a nuance that game-style VR might not provide effectively. Although game-style VR allows for an interactive learning process by grabbing and manipulating PPE in virtual space through the movement of controllers, it does not facilitate the learning of detailed hand movements. The immersive 360° VR tool does not require the use of controllers, allowing learners to mimic the instructor’s precise hand motion during training. For these reasons, the ability to develop low-cost, versatile VR content for teaching a wide range of medical procedures is important, making immersive 360° VR a superior option to game-style VR. Further research is needed to determine the skill retention and cost-effectiveness associated with immersive 360°VR vs game-style VR. Findings from such research may elucidate the optimal strategies for implementing VR in essential medical procedure education.

In the present trial, no significant difference in learning effectiveness was observed between the VR training and conventional face-to-face training for PPE donning and doffing procedures. To our knowledge, this study was the first to demonstrate the equivalency in effectiveness of this particular technique. Previous studies have indicated that the degree of immersion in a VR experience substantially increases the memorability of the experience.^30,31^ The immersive 360° VR system used in this study was based on high-quality 8000 resolution 360° video footage, allowing users to freely choose from 3 perspectives, including the instructor’s perspective, to simulate the experience of precise hand movements. In addition, high-quality audio was recorded to enhance auditory immersion so that the viewer could experience realistic sounds corresponding to each movement of PPE donning and doffing, such as the sound of hand hygiene and gown roll up. This realism may be the reason that the 360° videos used for VR simulated accurate handling with the same level of skill retention as face-to-face training that used actual PPE in the present study. A current limitation of VR is the challenge of accurately replicating tactile feedback within the VR experience, which poses a barrier to the acquisition of medical skills for which tactile sensation is paramount.

The learning effectiveness of VR training and face-to-face training was comparable and significantly higher than that of video training. Several studies on PPE donning and doffing techniques using video training suggest effective learning methods for health care professionals.^32,33,34^ On the other hand, another study has reported that, although video training is effective for learning clinical skills in the Objective Structured Clinical Examination, some students do not achieve adequate understanding due to the lack of interactivity.^35^ The video materials used in this study have been used for many years at Teikyo University Hospital. The video material was the standard content considered to be valid and reliable as teaching material in Japan. Content of the training was identical for the VR, face-to-face, and video groups. However, despite the identical content, video training was found to have significantly lower performance on procedures requiring fine motor skills when compared with VR and face-to-face training. The outcomes of this trial suggest that video training is not an appropriate method for untrained student health care professionals. Since the effectiveness of VR may vary depending on the subject, further research should be conducted with different subjects.

In this trial, performance variability within the VR group paralleled the variability observed in face-to-face and video groups. Practical skills test scores ranged from a high of 20.00 to a low of 11.00 points, with a mean (SD) of 17.70 (2.10) points. This score reflects the complexity and diversity of learning outcomes in VR training. Student learning efficacy in medical education is affected by a broad spectrum of factors, including learning experiences, assessment efficacy, emotional and socioenvironmental influences, organizational dynamics, and educational content relevance.^36,37,38^ Past studies in VR technology’s role in medical education highlight its varying effectiveness. Kim and Kim^39^ in 2023 found that less immersive VR environments may enhance knowledge outcomes, while Huang et al^40^ in 2016 noted the benefits of immersive and imaginative VR elements. Alharbi et al^41^ in 2020 and Zhao et al^42^ in 2021 reported higher knowledge retention and superior performance, respectively, in students with VR training. These findings suggest that VR’s design and individual learner differences affect learning effectiveness. The present study, while not delving into these specific factors, underscores the need for continued research to elucidate and optimize VR training’s effect on medical education. Future studies should focus on these factors to enhance VR training’s effectiveness and applicability.

The immersive 360° VR system developed for this trial,^28^ which is freely available to anyone with a VR head-mounted display, presents an economical training option. A head-mounted display can be purchased for approximately $400.00, but as the user base expands, the cost per user decreases, potentially reaching as low as $4.00 per person for 100 users and $0.40 per person for 1000 users. This cost contrasts with that for face-to-face training, which costs $5.30 per person for PPE used during donning and doffing training, in addition to instructor labor costs of approximately $40.00 per hour; the costs increase as the number of trainees increases. Although video training involves minimal additional costs, requiring only a standard personal computer or tablet, its effectiveness is often limited. Based on the trial findings and reports from existing literature, we believe that immersive 360° VR training offers a cost-effective and efficient method for training health care professionals, especially during pandemics. The affordability and higher efficacy of VR training compared with video-based methods make it a viable modality in the evolving realm of remote medical education. However, a comprehensive evaluation of these methods, focusing on long-term skill retention and broader applicability in various health care scenarios, is still needed.

### Limitations

This trial has several limitations. First, it was conducted at a single institution and included only medicine, medical technology, and pharmacy students. Second, the study’s focus on a younger demographic group raises questions about its applicability to a wider age range in health care. The influence of participants’ potential familiarity with VR, independent of gaming experience, needs further examination. Future research should include diverse age groups to confirm that the findings are applicable to the broader health care sector. Third, the study assessed only short-term retention of PPE donning and doffing procedures. Therefore, it is uncertain whether retention of skills is transferable in the long-term in clinical situations. Overall, further investigation is required to determine the long-term efficacy of VR training and its effect on clinical outcomes.

## Conclusions

In this trial, both the VR and face-to-face groups significantly outperformed the video group in PPE donning and doffing procedures. Furthermore, the video training group demonstrated significantly less accuracy in glove removal, hand hygiene, gown removal, and gown roll up. Overall, the findings offer valuable insights into the effectiveness of VR training in PPE donning and doffing procedures. With VR training’s ability to offer flexible learning experiences unbound by time and space, it is anticipated to be highly beneficial in both normal and pandemic situations. The findings also underline the potential of VR training to be a viable resource-conserving training option for PPE donning and doffing and to serve as a robust tool even for untrained student health care practitioners.

## Notes

Supplement 1.
Trial Protocol

Supplement 2. **eFigure.** Protocol of the Current Study

Supplement 3.
Data Sharing Statement
