# Supplementary material for: Effectiveness of Virtual Reality Training in Teaching Personal Protective Equipment Skills: A Randomized Clinical Trial
Source: JAMA Netw Open. 2024 Feb 14;7(2):e2355358. doi: 10.1001/jamanetworkopen.2023.55358 (PMC10867681; doi:10.1001/jamanetworkopen.2023.55358)
Supplement: Supplement 1. — Trial Protocol [file jamanetwopen-e2355358-s001.pdf]

Research Implementation Plan

1 Title

Evaluating the Effectiveness of a Virtual Reality (VR) Training Tool for Teaching Personal Protective Equipment (PPE) Donning and Doffing

2 Significance (Background) and Objectives

The COVID-19 pandemic continues to wreak havoc worldwide, and a multitude of infection control measures are being implemented from various angles. In healthcare settings, healthcare workers wear Personal Protective Equipment (PPE), such as masks, gowns, and gloves, to prevent infections. However, reports of cluster infections within medical facilities continue to surface. One reason cited for this is the lack of adherence to proper PPE donning and doffing techniques, particularly among healthcare workers with fewer years of experience. This issue has been suggested as one of the factors contributing to the emergence of clusters caused by multi-drug resistant bacteria. Traditionally, medical education and training for healthcare workers on "proper PPE techniques" have been conducted using physical materials, but this is becoming challenging due to the current shortage of medical supplies. While online texts and videos are being used as alternatives for hands-on training, these methods have not been as effective as practical experience, leaving gaps in the skills acquired by students and healthcare professionals.

In recent years, advances in digital technology have made it possible to create Virtual Reality (VR) images from high-quality 360-degree camera footage. Not only has VR begun to penetrate various fields, including entertainment and business, but it is also being applied as an educational and training tool in workplaces and schools. In the medical field, it has begun to serve as a simulation tool for surgeries.

The objective of this study is to develop a VR-based training tool for PPE donning and doffing (VR Training Tool) that is as effective as traditional hands-on training methods, in a context where medical supplies are limited. We aim to elucidate the usefulness of this VR training tool in acquiring proper PPE techniques. By doing so, we hope to establish a method for skill acquisition that doesn't consume limited medical resources and can be easily and conveniently used anytime, serving as an in-hospital infection control measure against COVID-19 and multi-drug resistant bacteria.

3 Research Methods

To clarify the utility of the VR educational training tool, we will conduct two types of trials: [1] a non-inferiority trial comparing the effectiveness of traditional Face-to-Face Training and VR Training, and [2] a three-group comparative trial among traditional Face-to-Face Training, VR Training, and 2D Video Training. Both trials will be PROBE trials (Prospective Randomized Open Blinded End-Point

Studies), where the randomization results will only be concealed from evaluators and analysts.

The study population will consist of university students from our institution who have not previously attended lectures on proper PPE donning and doffing techniques. Participants will be randomly divided into three groups: the Face-to-Face Training Group (classroom instruction + face-to-face donning and doffing practice), the VR Training Group (classroom instruction + simulated experience of donning and doffing using the VR training tool), and the 2D Video Training Group (classroom instruction + viewing of donning and doffing through a 2D educational video). Educational training will be conducted in these three groups, and the level of skill acquisition will be assessed through a practical skills test in PPE donning and doffing. The practical skills test will be administered to all participants three days after the educational training session. Statistical analyses will be conducted on the scores from the practical skills tests to identify differences in skill acquisition among the Face-to-Face Training Group, the VR Training Group, and the 2D Video Training Group.

【Development of the VR Educational Training Tool】

The applicants, in collaboration with Alpha Code Corp., a company with a track record in developing VR content, commenced the development of the VR educational training tool in September 2020. In November 2020, demonstrations of both the correct and incorrect methods of donning and doffing PPE were filmed using three high-quality 360-degree cameras. These demonstrations were conducted by PPE instructors affiliated with our university hospital, and explanatory audio was recorded. Based on this recorded data, a VR educational training tool was developed that can be experienced via a Head-Mounted Display (HMD) or smartphone. The tool was completed in January 2021.

＜Overview of the VR Educational Training Tool＞

[1] Filming Environment
Alpha Code Corp., Filming Studio (dimensions: 2.8m x 2.8m)

[2] Equipment Used
360-degree cameras (QooCam 8K, KanDao Technology Co), 3 units

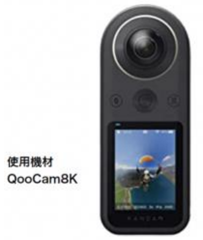

[3] Medical Materials Used

Gowns, gloves, surgical masks, alcohol disinfectant gel, waste containers

[4] Layout Diagram

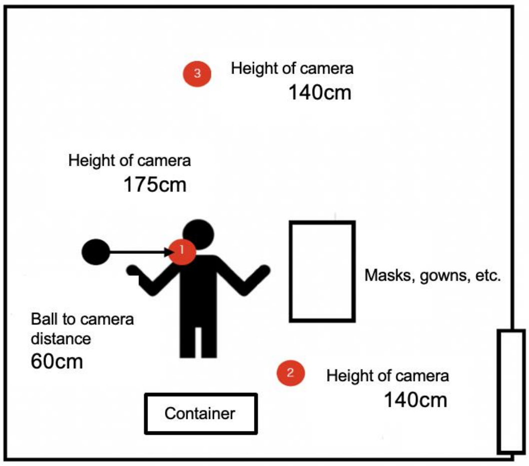

[5] Multi-Angle Footage

By placing 360-degree cameras at the instructor's left front, overhead, and rear positions, we enable participants to freely select their viewpoint for observing the procedures.

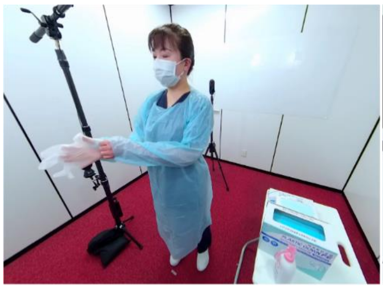

Front View

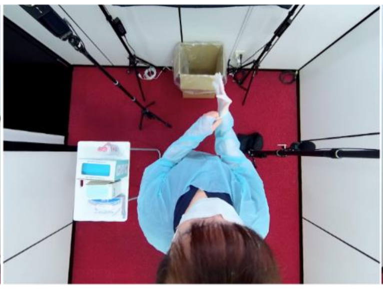

First-Person View

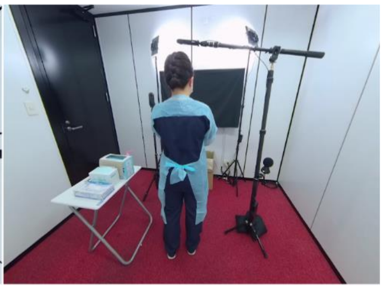

Rear View

[6] Procedure and Explanation for Donning and Doffing

Please refer to Attachment 1 for the procedure and explanation of donning and doffing. These were created under the supervision of the Infection Control Department of Teikyo University Hospital and include audio commentary.

[7] Head Mounted Displays Used

For VR: Pico G2 4K, Oculus Quest

＜Overview of the 2D Educational Training Video＞

The 2D training video has been produced by the Infection Control Department of Teikyo University Hospital and is already in use for training healthcare workers and medical students affiliated with the hospital on PPE donning and doffing.

＜Participants＞

The study will involve volunteers from the Itabashi Campus of Teikyo University who have consented to participate. There will be no restrictions on academic department. Recruitment will be conducted through emails to students, classroom announcements, and the posting of recruitment posters.

<u>Eligibility Criteria:</u>

Participants must meet the following conditions:

(A) Age 20 or older

(B) No gender restrictions

(C) Sufficient cognitive and decision-making abilities to understand the research study

<u>Exclusion Criteria</u>

(A) Individuals who have previously experienced discomfort or other adverse effects while using a head-mounted display for VR experiences

(B) Individuals who cannot give consent to participate in the research

(C) Individuals who have difficulty donning and doffing PPE

＜Data Collection Method＞

[1] At the time when the study participants have assembled, they will be randomly divided into three groups using a computer. The randomization of participants will be handled by the co-researcher from the Department of Orthopedic Surgery, Wataru Miyamoto. The results of the randomization will be managed by Wataru Miyamoto and shared with the co-researchers responsible for the educational training (Keisuke Tsukada from the Department of Orthopedic Surgery, Junko Fuyumuro and Tomomi Kikuchi from the Infection Control Department). The results will be used in the training. The principal investigator responsible for the skill test evaluation (Youichi Yasui from the Department of Orthopedic Surgery) will be blinded to the randomization results. Additionally, the co-researcher responsible for result analysis (Satoshi Miyata from the Department of Public Health) will also be blinded to the randomization, but will be informed about the assigned groups (A, B, C) for use in the analysis.

- Face-to-Face Training Group (lecture + practice using real materials): 30 participants
- VR Training Group (lecture + simulated experience using VR educational tools): 30 participants
- 2D Video Training Group (lecture + viewing method of donning and doffing using 2D educational video): 30 participants

[2] The data collection will take place in classrooms at the Teikyo University School of Medicine.

[3] Training days for donning and doffing methods, as well as days for conducting the skill test, will be set individually for each participant. To avoid crowding, the number of participants in each training session will be limited to 30 or fewer. Scheduling for data collection will be individually coordinated with each study participant.

[4] Educational training regarding donning and doffing methods will be conducted (Items 1 and 2 are carried out on the same day).

1) Lecture-based Training:

A 30-minute lecture-based training on PPE donning and doffing methods will be provided to all participants, conducted outside of regular lecture and practice hours. Seating will be arranged to avoid close contact among participants. The training will be managed by the co-researchers from the Department of Orthopedic Surgery (Keisuke Tsukada), the Infection Control Department (Junko Fuyumuro, and Tomomi Kikuchi).

2) Practical Training:

Following the end of Item 1, 30 minutes of practical training will be given for each group using real PPE, VR educational training tools, or 2D educational training videos. Separate classrooms will be prepared for each group to prevent cross-contamination. Face-to-face instruction and management of VR and 2D training tools will be handled by the co-researchers mentioned above.

[5] A practical skills test on PPE donning and doffing methods will be conducted individually for all participants three days following their respective educational training sessions. To ensure evaluation accuracy and to prevent any influence from the evaluator's movements on the participants' donning and doffing actions, the entire sequence will be video-recorded from three different angles for later evaluation. The estimated time required for this is approximately 10 minutes. The test will take place in classrooms at Teikyo University School of Medicine. The test will be evaluated by the principal investigator, Youichi Yasui from the Department of Orthopedic Surgery, who is not involved in randomization or educational training. Videos used

for evaluation will conceal the participants' group affiliations and will be played back and evaluated in random order.

[6] The following data will be collected:
- Personal information (Name, Age, Gender)
- Video recordings of the practical skills test on donning and doffing methods
- Evaluation scores of the practical skills test (Skill level acquisition: refer to Supplementary Material 2)

The evaluation will be based on a 20-item, 20-point scale that measures whether the procedure was conducted correctly, whether hand sanitization was carried out, and whether each piece of PPE was donned and doffed accurately.

＜Evaluation Criteria＞

The evaluation criteria will be as follows:

□ Primary evaluation criteria: Practical skills test scores of the Face-to-Face Training Group and the VR Training Group

□ Secondary evaluation criteria: Practical skills test scores of the Face-to-Face Training Group, VR Training Group, and the 2D Video Training Group.

＜Statistical Analysis＞

The current study will employ an Intention to Treat (ITT) analysis. Statistical analysis will be conducted by a research collaborator not involved in randomization or educational training, Satoshi Miyata from the Graduate School of Public Health.

Primary Analysis Method: A non-inferiority test will be performed based on the mean scores and variances of the Face-to-Face Training Group and the VR Training Group. T-tests and a comparison of the 95% confidence intervals of the mean scores for each group will be used to demonstrate that the VR Training Group is not inferior to the Face-to-Face Training Group.

Secondary Analysis Method: A variance analysis and multiple comparisons using Tukey's method will be conducted based on the mean scores and variances of the Face-to-Face Training Group, VR Training Group, and the 2D Video Training Group, in order to reveal any differences in the educational training effects among the three groups.

4 Target Sample Size

Total Target Participants: 90 cases

Face-to-Face Training Group: 30 cases

VR Training Group: 30 cases

2D Video Training Group: 30 cases

Rationale for Setting: The sample size is designed based on the results of a pilot study conducted in our department on the skill acquisition of PPE donning and doffing (Verification of Educational Training Effectiveness on PPE Donning and Doffing Techniques 20-240), which yielded an mean score of 17.7 and a variance of 3.01. For the primary endpoints in this trial, calculations were made with a one-sided significance level of 0.025, a power of 0.8, and an Effect size of -1.7, yielding a sample size of n=16.3 (for the non-inferiority test) and n=21.9 (for the equivalence test) for each group. For the secondary endpoints, the sample size was calculated to be n=28.9 based on the estimated mean scores for each group (Face-to-Face Training Group: 17.7, VR Training Group: 16, 2D Video Training Group: 14), with a significance level of 0.01 and a power of 0.8. Considering the potential for ineligible cases to be identified after registration, the sample size for each group was set to n=30. The sample size setting was conducted by the statistical coordinator, Satoshi Miyata from the Graduate School of Public Health.

5 Research Organization

(○ Principal Investigator at our University, ◎ Data Management Responsible Person at our University):

Principal Investigator (Data Management Responsible)

○ Yoichi Yasui, Department of Orthopedic Surgery, Faculty of Medicine, Teikyo University, Lecturer

Co-Investigators:

◎ Wataru Miyamoto, Department of Orthopedic Surgery, Faculty of Medicine, Teikyo University, Lecturer

Keisuke Tsukada, Graduate School of Medicine, Teikyo University, Graduate Student

Seikai Toyooka, Graduate School of Medicine, Teikyo University, Graduate Student

Junko Fuyumuro, Infection Control Department, Teikyo University Hospital, Head Nurse

Tomomi Kikuchi, Infection Control Department, Teikyo University Hospital, Associate Director

Satoshi Miyata, Graduate School of Public Health, Teikyo University, Professor

6　Study Period

June 9, 2021 to March 31, 2023.

7　Ethical Considerations in Research

This study will be conducted in accordance with the "Ethical Principles Based on the Declaration of Helsinki" and the "Ethical Guidelines for Medical Research Involving Human Subjects." We will adhere to this research plan throughout the study.

1) Informed Consent

The acquisition of consent for participation in the study will be conducted at the Orthopedic Outpatient Clinic of Teikyo University Hospital using study explanatory documents, consent forms, and withdrawal forms approved by the Teikyo University Medical Research Ethics Committee. This will be executed by the principal investigator and the co-investigators.

The content to be explained is as follows:

The purpose and significance of the study, the subjects and methods involved, the freedom to voluntarily participate or withdraw consent, the responsible individuals and organizations involved, the location and duration of the study, the handling of research samples and information, the treatment of research outcomes, funding sources, conflicts of interest, whether there will be any burden or payment for participants, potential benefits and risks to individuals, adverse events and how they will be addressed, conditions for halting the study, and how to address questions and who to contact.

Participants will be informed that they have the freedom to participate, withdraw consent, and will not suffer any disadvantage due to non-participation or withdrawal of consent. It will be explained that withdrawal of consent will be accepted at any time upon submission of a written form.

Consent and withdrawal of consent will be obtained via signatures from the study subjects. For questions, access to documents, or non-participation, Yoichi Yasui, lecturer in the Department of Orthopedic Surgery at Teikyo University's School of Medicine (contact phone number: +81-3-3964-1211), will be responsible for responding.

2) Methods for Storage and Disposal of Information (Including Materials Related to Information Used in Research)

Access to information about study participants will be conducted at the Department of Orthopedic Surgery at Teikyo University School of Medicine. Only anonymized information will be stored on

the co-investigators' computers. Information that can identify individuals will be removed from the original data, and new ID numbers will be assigned to anonymize it. A correspondence table between individual names and ID numbers will be created.

The correspondence table and the computer used for analysis will be stored in the Orthopedic Surgery booth within the Frontier Research Unit (hereafter referred to as FRU) at Teikyo University. Paper media and video data will be stored in a lockable desk assigned to co-investigator Wataru Miyamoto of the Department of Orthopedic Surgery, and the computer will be locked when not in use and stored in a lockable cabinet within the FRU Orthopedic Surgery booth. Randomization results recorded in the allocation table will be stored in a lockable cabinet in the orthopedic medical office.

The person responsible for handling the original data, correspondence table, and allocation table will be co-investigator Wataru Miyamoto of the Department of Orthopedic Surgery. Access to the original data and the correspondence table will be limited to the principal investigator and co-investigators. Access to the allocation table will be restricted to four individuals, including the co-investigator responsible for randomization (Wataru Miyamoto) and those in charge of educational training (Keisuke Tsukada, Junko Fuyumuro, and Tomomi Kikuchi).

Information about individuals who withdraw consent will be immediately destroyed. The information of the study participants will not be used for purposes other than the research objective. After the conclusion of the study, the information will be stored for 10 years following the publication of the respective paper or other output, based on the "Guidelines for the Preservation of Research Data at Teikyo University and Teikyo Junior College." For digital data, appropriate metadata management and backups will be created to make the data reusable.

For inquiries or complaints regarding stored personal information, participants can contact the Department of Orthopedic Surgery at Teikyo University School of Medicine (main telephone: +81-3-3964-1211), where they will receive assistance from Yoichi Yasui, a lecturer in the Department of Orthopedic Surgery.

Information obtained in this study may potentially be reused for other research to be conducted in the future by the Department of Orthopedic Surgery at Teikyo University School of Medicine. If information is to be reused for other research, the study will undergo a separate ethical review process before being conducted.

3) Methods for Sample Storage and Disposal

Not Applicable

8 Potential Risks and Inevitable Discomforts (Adverse Events) from Participation in the Study

In this study, participants in the VR Training Group will view and experience educational content on PPE donning and doffing using a commonly used VR Head Mounted Display. While care will be

taken to prevent incidents like falls for safety reasons, if a participant feels uncomfortable and finds it difficult to continue while viewing or experiencing the VR content, the viewing or experience will be immediately halted, and a research team member will respond accordingly.
Similarly, although the likelihood of experiencing discomfort during the PPE donning and doffing is extremely low, if such symptoms do occur, the activity will be immediately stopped, and an on-site research team member will take appropriate action.

Should medical treatment like intravenous drips be necessary, participants will be informed that the medical costs will be covered by their health insurance.

Since the study participants are students of this university, they will be informed that their decision to participate or not in the study will have no bearing on their academic evaluations at the university.

9 Benefits Arising from Participation in the Study

Upon completion of the study, participants will be informed of their performance on the practical test for PPE donning and doffing, and will receive suggestions for improvements and necessary advice. Each participant will also receive a monetary reward of 3,000 yen.

10 Conditions for Study Termination and Corresponding Measures

If a data breach occurs, the study will be immediately terminated, and the principal investigator will promptly report to the ethical committee and the study participants. Similarly, the study will be terminated if adverse events occur that are conclusively attributed to the study, or if the principal investigator and co-investigators determine that it is not appropriate to continue the study. Upon termination, prompt reporting to the ethical committee will be done, and participants will be informed via the website. Any results obtained up to the point of study termination will be securely stored for five years and then disposed of as previously stated.

11 Monitoring and Auditing

Not applicable.

12 Post-study Measures for Participants

After the study, participants will be informed of their results on the PPE donning and doffing practical test, and will receive suggestions for improvements and necessary advice.

13 Compilation and Handling of Research Outcomes

All data obtained as research outcomes will be treated as confidential. When publishing the research results, the data will be presented in aggregate form to ensure that individual participants cannot be identified. Research findings will be reported in academic conferences and journal articles. Care will

be taken to protect individual information during this process.

14 Source of Research Funding

The research will be conducted using funds from the Department of Orthopedic Surgery at Teikyo University School of Medicine.

15 Conflict of Interest

For this study, we have received financial support from Alpha Code Corporation for the production of the VR educational tool.

16 Interim and Final Reporting to the Ethics Committee

Interim reports, which include details on the progress (number of cases), ethical considerations, adverse events, and research findings, will be submitted to the Ethics Committee Secretariat annually. Additionally, a final report will be submitted upon the completion of the study.

17 List of Supplementary Materials

Document 1: Donning and Doffing Procedure

Document 2: Evaluation Criteria

Document 1: Donning and Doffing Procedure

1 How to put on and take off personal protective equipment - standard

2 Here's how to put on personal protective equipment.

3 First, disinfect your hands.

4 Push the disinfectant pump all the way down and put a sufficient amount into your palm.

5 Rub it into your palms, fingertips, the backs of your hands, between your fingers, thumbs, and around your wrists.

6 The time that the disinfectant is in contact with the skin is important. Rub it in until it dries.

7 Next, put on the gown.

8 Take the gown out of the box and unfold it.

9 Put it around your neck, run the sleeves through, and place the thumb hook over your thumb.

10 Tie the waist strap at the back.

11 The waist strap can be tied in a bow or a knot.

12 Tie the knot in a way that's easy for you.

13 Next, put on the surgical mask.

14 Take the surgical mask out of the box and put it on with the nose piece upwards and the pleats downwards.

15 Fit the nosepiece to your nose and unfold the pleats to cover your nose to chin.

16 Finally, put on the gloves.

17 Take one glove out of the box and put it on.

18 With the gloved hand, take the other glove and put it on.

19 Make sure the gloves cover the cuffs of the gown so that your wrists are not exposed.

20 Now you're finished with putting on the personal protective equipment.

21 Here's how to remove personal protective equipment.

22 Pinch the outside of the glove and remove it while turning it inside out.

23 Roll up the removed glove and hold it in your gloved hand.

24 Remove the other glove by inserting your fingers into the gap by your wrist, and remove it without touching the outside of the glove.

25 Dispose of the two gloves in one go.

26 Next, disinfect your hands.

27 The gloves may have pinholes, or your hands may have touched the outside of the gloves when removing them, so be sure to disinfect your hands after removing the gloves.

28 At this point, it's OK just to disinfect any exposed parts of the body.

29 Next, take off your gown.

30 First, tear off the neck strap, then the waist strap.

31 Put your fingers inside one cuff and pull your arm out of the sleeve until it's about one palm length from the end.

32 Grasp the sleeve on the other side through the gown and pull your other arm out.

33 Then hold the gown away from you so that it does not touch your body or clothes, and dispose of it by rolling it up with the outside of the gown on the inside.

34 If you feel that your hands are contaminated in any way, disinfect them again.

35 Next, remove the mask.

36 To avoid touching the surface of the mask, remove it by holding the rubber strap, and dispose of it.

37 When disposing of personal protective equipment, make sure it goes inside the disposal container completely, and does not stick out.

38 Finally, disinfect your hands.

39 The time that the disinfectant is in contact with the skin is important. Rub it in until it dries.

40 Now you’re finished with taking off the personal protective equipment.

41 There are some mistakes in the steps you’re about to see.

42 See if you can spot them.

43 Could you tell what was wrong?

44 (1) Taking out the gown without disinfecting the hands.

45 He took the gown out of the box without disinfecting his hands.

46 If the gown is taken out without disinfecting the hands, the box will be contaminated.

47 Make sure to disinfect your hands first to prevent contamination.

48 (2) Not using the thumb hooks on the gown and not tying the waist strap.

49 If you don't use the thumb hooks, your wrists will be exposed.

50 To prevent contamination of the wrist, the thumb hook should always be placed over the thumb.

51 If the waist strap of the gown is not tied, the gown may come off during use, touching surrounding objects, and contaminating your own clothes.

52 Make sure to tie the gown’s waist strap.

53 (3) Putting on the gloves first, then the mask.

54 He put on the gloves before putting on the mask.

55 If you put on the gloves first, you’ll get your own germs on the gloves.

56 Gloves should be put on last to ensure that clean gloves are used with patients.

57 (4) Putting on the surgical mask without adjusting it.

58 He didn't fit the surgical mask properly, which left a gap.

59 To prevent exposure to your own nasal and oral mucosa, fit the nosepiece and unfold the pleats to cover the entire area from nose to chin.

60 Now, let's watch someone remove personal protective equipment.

61 There are some mistakes in the steps you are about to see.

62 See if you can spot them.

63 Could you tell what was wrong?

64 (1) After removing the gloves, taking off the gown without disinfecting the hands.

65 The gloves may have pinholes, or your hands may be contaminated by touching the outside of the gloves when removing them.

66 Make sure to disinfect your hands after removing the gloves.

67 (2) The gown was removed incorrectly, and part of the gown was sticking out from the disposal container.

68 As he removed his arms from the sleeves, his hands touched the surface of the contaminated gown.

69 The surface of the gown is contaminated. If you pull your arms straight out of the sleeves, your hands will be contaminated.

70 He also rolled up the gown on his body.

71 If you roll it up on your body, your clothes will be contaminated.

72 When taking off the gown, it's important to remove your arms from the sleeves so that your hands are not contaminated, and then roll up the gown with both hands extended so that it doesn't touch the body.

73 When disposing of personal protective equipment, make sure it doesn’t stick out from the disposal container.

74 (3) Holding the filter area of the surgical mask to remove it.

75 He grasped the filter area of the surgical mask and removed it.

76 The surface of the mask is contaminated.

77 To prevent hand contamination, make sure to remove it by holding the rubber strap, without touching the filter area.

78 (4) Not pushing the disinfectant pump all the way down, lightly spreading disinfectant over the hands, and shaking the hands to dry them.

79 The disinfectant pump is designed to release the proper amount when fully pushed down.

80 Don't shake or clap your hands. Rub in the disinfectant until it dries.

81 Now you’re finished with taking off the personal protective equipment.

Document 2: Evaluation Criteria

Donning PPE	
1	Perform hand hygiene
2	Sufficient hand hygiene was done
3	Put on a gown
4	The gown was put on correctly
5	Put on a surgical mask
6	The surgical mask was put on correctly
7	Put on gloves
8	The gloves were put on correctly
Doffing PPE	
1	Remove gloves
2	The gloves were removed correctly
3	Perform hand hygiene
4	Sufficient hand hygiene was done
5	Remove a gown
6	The gown sleeves were removed correctly
7	The gown roll up was correct
8	Remove a surgical mask
9	The surgical mask was removed correctly
10	Perform hand hygiene
11	Sufficient hand hygiene was done
12	The PPE disposal was accurate
