# Supplementary material for: Effectiveness of Virtual Reality Training in Teaching Personal Protective Equipment Skills: A Randomized Clinical Trial
Source: JAMA Netw Open. 2024 Feb 14;7(2):e2355358. doi: 10.1001/jamanetworkopen.2023.55358 (PMC10867681; doi:10.1001/jamanetworkopen.2023.55358)
Supplement: Supplement 2. — eFigure. Protocol of the Current Study [file jamanetwopen-e2355358-s002.pdf]

Supplemental Online Content

Tsukada K, Yasui Y, Miyata S, et al. Effectiveness of virtual reality training in teaching personal protective equipment skills: a randomized clinical trial. *JAMA Netw Open*. 2024;7(2):e2355358. doi:10.1001/jamanetworkopen.2023.55358

eFigure. Protocol of the Current Study

This supplemental material has been provided by the authors to give readers additional information about their work.

© 2024 Tsukada K et al. *JAMA Network Open*

eFigure. Protocol of the Current Study

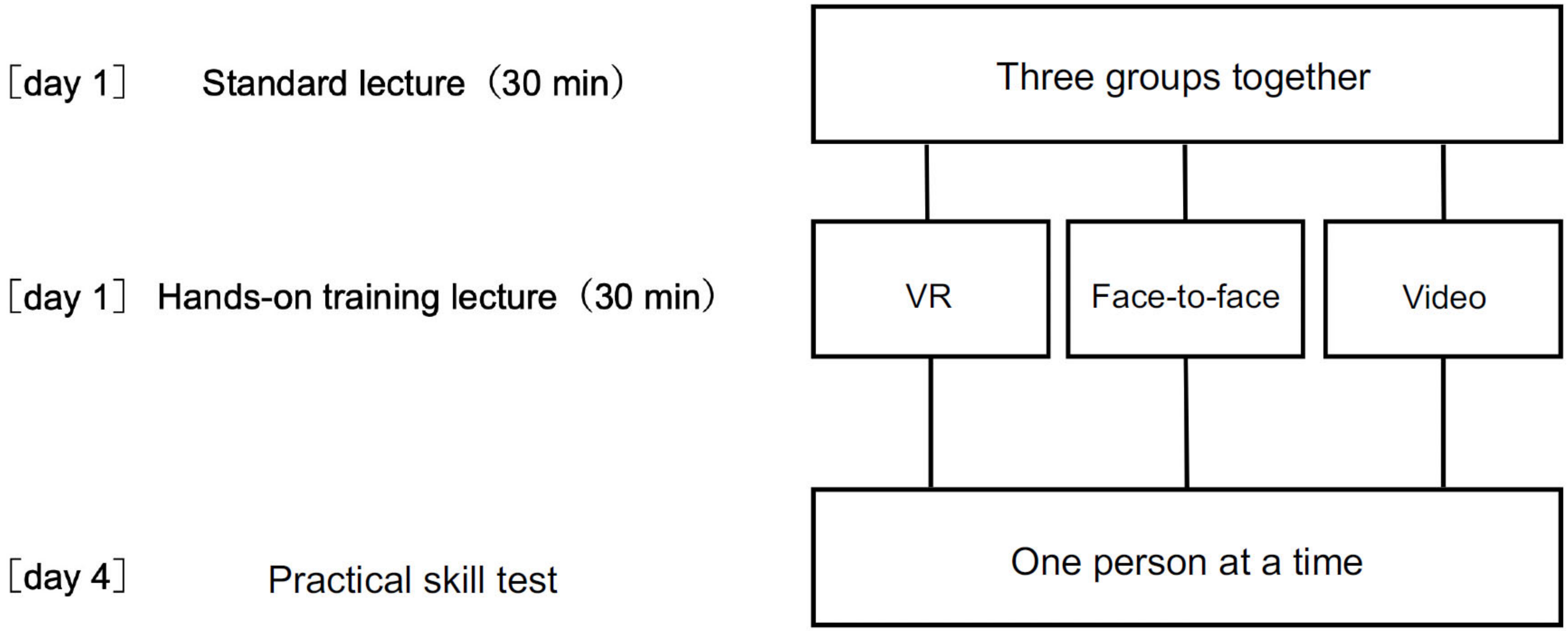

© 2024 Tsukada K et al. *JAMA Network Open*
