# Supplementary material for: Effectiveness of Virtual Reality Training in Teaching Personal Protective Equipment Skills: A Randomized Clinical Trial
Source: JAMA Netw Open. 2024 Feb 14;7(2):e2355358. doi: 10.1001/jamanetworkopen.2023.55358 (PMC10867681; doi:10.1001/jamanetworkopen.2023.55358)
Supplement: Supplement 3. — Data Sharing Statement [file jamanetwopen-e2355358-s003.pdf]

Data Sharing Statement

Tsukada. Effectiveness of Virtual Reality Training in Teaching Personal Protective Equipment Skills. *JAMA Netw Open*. Published February 14, 2024.
doi:10.1001/jamanetworkopen.2023.55358

Data
Data available: Yes
Data types: Deidentified participant data, Other (please specify)
Additional Information: Immersive 360° VR Tool
How to access data: Deidentified participant data: Please send your request via email. The email address is, "k.r.k.tsuka@gmail.com". Immersive 360°VR Tool: https://share.blinky.jp/s/NDUwNA
When available: With publication

Supporting Documents
Document types: Statistical/analytic code, Informed consent form
How to access documents: Please send your request via email. The email address is, "k.r.k.tsuka@gmail.com".
When available: With publication

Additional Information
Who can access the data: Anyone requesting the data.
Types of analyses: For any purpose.
Mechanisms of data availability: After approval of a proposal.
